# Neonicotinoids suppress contact chemoreception in a common farmland spider

**DOI:** 10.1038/s41598-020-63955-z

**Published:** 2020-04-27

**Authors:** Stanislav Korenko, Jakub Sýkora, Milan Řezáč, Petr Heneberg

**Affiliations:** 1 Czech University of Life Sciences Prague, Faculty of Agrobiology, Food and Natural Resources, Department of Agroecology and Crop Production, Prague, Czech Republic; 2 Crop Research Institute, Biodiversity Lab, Prague, Czech Republic; 3 Charles University, Third Faculty of Medicine, Prague, Czech Republic

**Keywords:** Entomology, Animal behaviour, Natural hazards

## Abstract

Neonicotinoid insecticides are increasingly recognized for their role as information disruptors by modifying the chemical communication system of insects and therefore decreasing the chances of reproduction in target insects. However, data from spiders are lacking. In the present study, we tested the responses of males of a common agrobiont spider, *Pardosa agrestis*, to the application of field-realistic concentration of acetamiprid, which was formulated as Mospilan, and trace amounts of thiacloprid, which was formulated as Biscaya. We applied fresh or 24-h-old residues of Mospilan or Biscaya to the males just prior to the experiment or treated only the surface of a tunnel containing female draglines. We evaluated the ability of the males to recognize female cues from female dragline silk in a Y-maze. The field-realistic, sublethal doses of Mospilan altered pheromone-guided behavior. The choice of the tunnel with female draglines by males was hampered by tarsal treatment of the males with 24 h-old residues of Mospilan. The mating dance display was commonly initiated in control males that came into contact with female draglines and was suppressed by the Mospilan treatments in all three experimental settings. Some males only initiated the mating dance but did not manage to complete it; this was particularly true for males that were treated tarsally with fresh Mospilan residues, as none of these males managed to complete the mating dance. All three experimental settings with Mospilan decreased the frequency of males that managed to both select the tunnel with female draglines and complete the mating dance. The responses to the low-dose Biscaya were much milder and the study was not sufficiently powered to confirm the effects of Biscaya; however, the surprisingly observed trends in responses to very low Biscaya concentrations call for further analyses of long-term effects of trace amounts of neonicotinoids on the pheromone-guided behavior of spiders. These are the first conclusive data regarding the effects of commercially available formulations of neonicotinoid insecticides on the intraspecific chemical communication of spiders.

## Introduction

Chemical signals are crucial for spider reproduction because they allow the location of mates. In spiders, one of the potential sources of chemical signals is female silk, which attracts mates and elicits courtship^1–4^. The wandering spiders use silk draglines for mate searching and mate attraction^5^, male courtship^6,7^ and the evaluation of mating status^8^. Contact chemoreception typically involves compounds of large molecular weight that remain stable and accessible to receivers long after the sender has left the area, such as long-chain hydrocarbons, fatty acids, lipids, and proteins^9^. In spiders, contact chemoreception involves tip-pore sensilla located on the legs and pedipalps^10–12^. When contacting the silk (or cuticle) of a female, these chemosensory setae elicit male courtship behaviors^13–15^. The chosen model species, *Pardosa agrestis* (Westring, 1861) (Araneae: Lycosidae), is unable to communicate via airborne cues, which distinguishes *P. agrestis* from other species of the same genus, such as *Pardosa milvina* (Hentz, 1844)^16^. Instead, the males of *P. agrestis* were previously shown to prefer paths covered with female silk, and this behavior has been disrupted by the exposure of the male spiders to the herbicide glyphosate and to the combination of the insecticides chlorpyrifos and cypermethrin^17^.

Neonicotinoids are compounds that act as potent insecticides, but their application may affect other nontarget organisms, such as spiders^18^. Neonicotinoids impair the nanostructural, chemical and mechanical properties of the silk itself^19^ and induce the temporary paralysis of spiders^18^. Yellow pan traps with aqueous solutions of imidacloprid were shown to lead to higher capture rates of spiders^20^. The effects of neonicotinoids on contact chemoreception, male attraction and courtship behavior are unknown.

Based on our preliminary observations, we hypothesized that contact chemoreception in spiders is affected by treatment with neonicotinoids. We tested whether dragline identification, mating dance initiation and mating dance completion are affected by treatment with neonicotinoids. As the mechanisms of impairment were unknown and the literature provided no indices, we applied three modes of exposure to neonicotinoids: a) males directly exposed for one hour prior to the experiment, b) males exposed for one hour to 24 h-old residues of neonicotinoids prior to the experiment, and c) application of neonicotinoids only to the bottom of the maze along a corridor that contained attached female draglines.

## Materials and Methods

### Collection and rearing of spiders

We collected juveniles of *P. agrestis* (n = 160) at the edge of a barley field in the Prague-Suchdol environment, Czechia (50°08′15″N,14°21′22″E, 301 m a.s.l.) in March and April 2018. We kept the spiders individually in glass tubes of 15 mm in diameter and 60 mm in length equipped with a layer of plaster of Paris at the bottom. We moistened the plaster of Paris with several drops of water every third day to maintain adequate humidity and reared the spiders until they reached adulthood. We kept the spiders at 20–22 °C under a natural photoperiod and fed them with wingless fruit flies *Drosophila melanogaster* Meigen, 1830 and juvenile cockroaches *Blatta lateralis* Walker, 1868.

### Establishment of experimental groups

**Figure 1:**
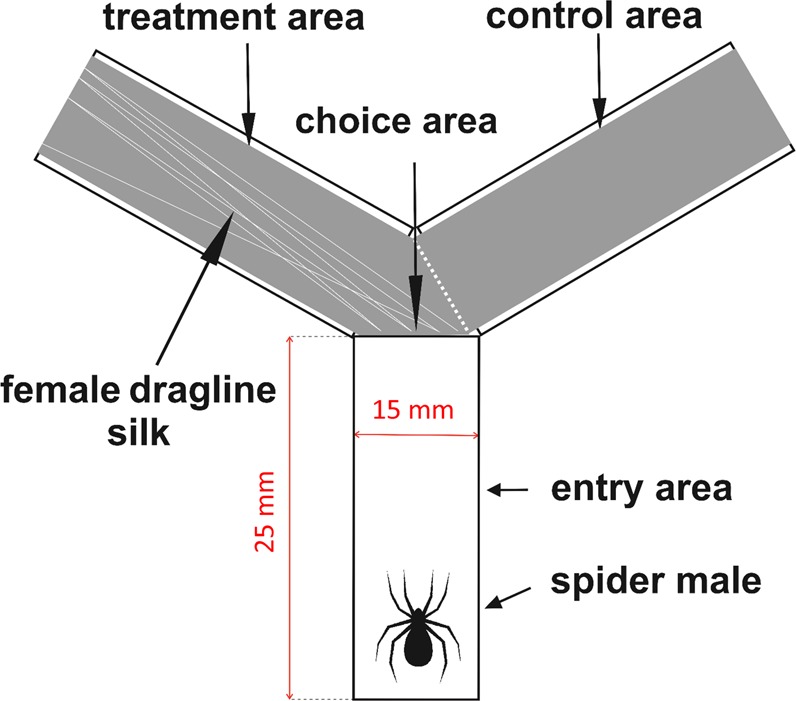
Scheme of the experimental maze used in the present study.

We conducted all the experiments only with adult virgin females and males and excluded those adults that displayed any injuries. The total number of individuals that fulfilled the above conditions was 144. The experiment consisted of testing the ability of males to recognize female cues from female dragline silk in the Y-maze. We designed the Y-maze from three plastic corridors of 25 × 15 × 10 mm (length x width x height) in size. The top parts of the corridors were covered with an 85 × 65 mm plastic lid to allow access of the experimentator. The corridors were arranged in a Y shape; therefore, the entrance area bifurcated in the so-called choice area into two corridors, one of which was provided with the draglines, and another was free of them (Fig. 1).

We obtained the draglines from virgin females as specified below. Concerning the males, we split them into four groups that were used for 1) an experiment with spiders treated for one hour tarsally with fresh residues of neonicotinoids, 2) an experiment with spiders treated for one hour tarsally with 24 h-old residues of neonicotinoids, 3) an experiment in which only the corridor with draglines was treated with fresh residues of neonicotinoids, and 4) a control experiment with spiders treated for one hour tarsally with distilled water. We conducted all experiments with two parallel groups and employed two different neonicotinoids as specified below. We used four groups of 12 females to prepare the draglines, and we assigned 12–15 males to each neonicotid-treated parallel group (for a total of six groups). We also formed a control group that consisted of 22 males.

### Collection of dragline silk fibers

Draglines, the product of the major ampullate silk glands, are known to carry contact sexual pheromones^17^. We collected dragline fibers from virgin adult females by placing them individually in Petri dishes 150 mm in diameter. The base of the Petri dishes was equipped with black paper, to which the females attached the draglines. After 60 mins of spider activity, we removed the draglines with tweezers and connected ten to fifteen draglines into lines spread longitudinally on Y-shaped black paper. Prior the attachment of draglines, we treated part of the Y-shaped black paper with the tested compounds (treatment area and choice area were treated). The remaining part of the Y-shaped black paper (control area) was untreated with the tested compounds and was treated with distilled water as a sham control. Dotted line in Fig. 1 indicates the border between the treated and control areas. After attaching the draglines, we placed the Y-shaped black paper in the treatment area in such a way that the draglines were stretched across the whole length of one branch of the maze. Another branch of the maze was without any silk. The draglines extended across the choice area, where the males first came into contact with the female dragline silk and were allowed to decide whether to follow the draglines (Fig. 1).

### Recorded variables

We recorded the male choice (female silk tunnel/control tunnel), the presence of a mating dance (at least an incomplete display), and the presence of a full (completed) mating dance in response to the contact chemical attractants. We followed the mating dance description provided by Kronestedt^11^ and Chiarle *et al*.^21^. In males of the study species, the mating dance is manifested by alternately raising and lowering the palps (up-down movements), while the cymbium draws semicircles with the palps (side-to-side movements). Visual stimulus from contact with the female was not provided; therefore, the above-described dance sequence was considered complete. In the case of visual contact, the males would perform the follow-up behavioral sequences that consist of raising the forelegs and their slight back-and-forth movements in synchrony with the movements of the palps and the abdomen. The spiders displayed this behavior only rarely, and we did not include it in the analyses. When up-down and side-to-side movements were displayed, we classified the mating dance as complete. When only weak up-down movements were displayed, we classified the mating dance as incomplete. We excluded all spiders that did not reach the choice area, where they would come into tarsal contact with female drag line silk. This typically happened when the spiders were climbing along the side walls or on the top lid as tarsal contact is considered mandatory to detect female cues from dragline silk in *P. agrestis* males^17^.

### Application of insecticides

We applied field-realistic concentrations of neonicotinoid insecticides in dilutions recommended by their manufacturers for use in spraying crops to eliminate pest insects (acetamiprid) or even by two orders below the recommended concentrations (thiacloprid). The tested insecticides consisted of the neonicotinoid acetamiprid (formulated as Mospilan 20 SP; dilution 25 mg L^−1^; treatment 19.9 μL cm^−2^ of the black paper, and 6.32 μL cm^−2^ of the filter paper, respectively) and another neonicotinoid thiacloprid (Biscaya 240 OD; dilution 360 μg L^−1^; treatment 19.9 μL cm^−2^ of the black paper, and 6.32 μL cm^−2^ of the filter paper, respectively). We excluded some other neonicotinoid insecticides, such as clothianidin, as these are used only for the treatment of seeds before they are sown. Note that some effects of the formulations used may have resulted from other compounds present in the commercially available formulations; therefore, we refer to their trade names rather to the names of active ingredients alone.

We used three application modes of the tested compounds or distilled water:

A) We applied the fresh residues of the tested formulations tarsally onto male spiders. To perform the tarsal application of fresh residues of the tested compounds, we rolled filter paper of 55 mm in diameter to form tubes of 55 mm in length and 10 mm in diameter and inserted these rolls into glass tubes of the same size. Next, we applied the tested formulations by injecting them at the surface of the filter paper. Next, we inserted males into the tubes, closed the tubes and kept the spiders in tarsal contact with the treated filter paper for one hour.
B) We applied the 24 h-old residues of the tested formulations tarsally onto male spiders. The experiment was designed as in (A); however, the tested compounds were deposited onto the filter papers 24 h prior to the experiment. Just before we deployed the spiders, we moistened the filter paper with distilled water at 6.32 μL cm^−2^ of the filter paper. Next, we inserted males into the tubes, closed the tubes and kept the spiders in tarsal contact with the treated filter paper for one hour.
C) We applied the formulations onto the black paper at the base of the tunnel where we later installed the female dragline silk. Therefore, to analyze the female cues, we treated the treatment area before the installation of female silk. We applied the tested formulations by spraying a controlled amount of the neonicotinoids in distilled water using the Potter Precision Laboratory Spray Tower (Burkard Scientific, Uxbridge, UK).

### Statistical analyses

We tested every treatment on at least 12 spider males; the control group consisted of 22 males. According to the power calculation (http://clincalc.com/stats/samplesize.aspx), these group sizes should allow the identification of a 40% decrease in the incidence of the analyzed types of behavior in comparison to that exhibited by the untreated population, which was expected to show 100% incidence of the analyzed types of behavior, assuming constant variance, α = 0.05 and 80% power. Data are shown as the frequencies within the treated groups. We used the Fisher exact test to analyze the differences in binary values. The spiders that did not come into contact with female draglines or that were inactive over the course of the experiment were excluded from the analyses. The analyses were conducted in SigmaPlot 12.0.

## Results

We found that both tested formulations of neonicotinoid insecticides had adverse effects on the chemical communication of *P. agrestis*. However, the effects were not identical when comparing the two tested formulations of neonicotinoid insecticides and the three modes of application.

**Figure 2:**
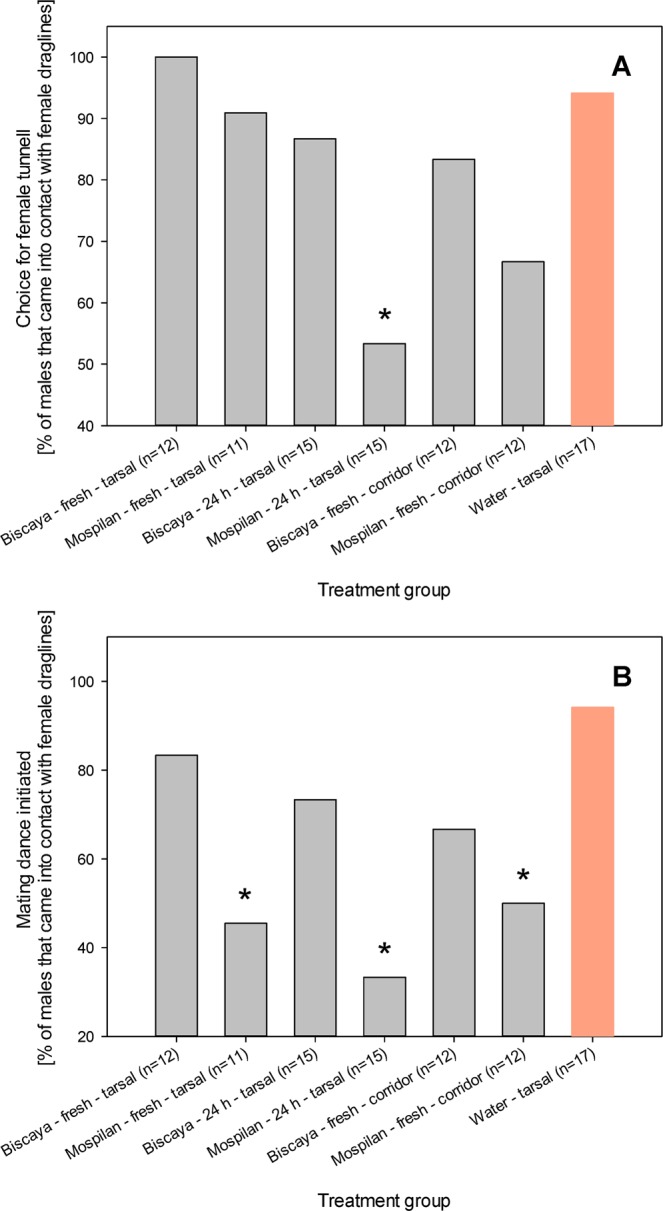
Effects of neonicotinoid insecticides on contact chemoreception of male *P. agrestis*. Frequencies of males that chose the tunnel with the presence of female draglines (**A**) and frequencies of males that initiated the mating dance in response to contact with female draglines (**B**). Numbers of tested individuals in each group are indicated; the provided numbers represent the numbers of males that came into contact with female draglines. Fisher exact test outcomes at *p* < 0.05 are indicated by * when compared to the water-treated group.

The choice of the tunnel with female draglines was commonly observed in control males that came into contact with female draglines (16/17 males). This behavior was not altered by the tarsal treatment of males with Biscaya, as both fresh and 24 h-old residues did not alter the frequency of males that chose the tunnel with the draglines (12/12 males and 13/15 males, respectively; Fisher exact test *p* > 0.05 each, d_f_ = 1) (Fig. 2A). Similarly, the treatment of the tunnel with the draglines with Biscaya did not alter the choice of the tunnel with female draglines (10/12 males; Fisher exact test *p* > 0.05, d_f_ = 1). In contrast, tarsal treatment with 24 h-old Mospilan residues prevented the males from choosing the tunnel with female draglines (8/15 males; Fisher exact test *p* = 0.01, d_f_ = 1). There was a trend towards a similar response when the tunnel with the draglines was treated with Mospilan; however, the study was not sufficiently powered to confirm this response (8/12 males; Fisher exact test *p* = 0.13, d_f_ = 1) (Fig. 2A).

The mating dance display was commonly initiated in control males that came into contact with female draglines (16/17 males). The effects of the Biscaya treatment were again less prominent compared to those of the Mospilan treatment and were not significant (10/12 males, 11/15 males and 8/12 males, respectively; Fisher exact test *p* = 0.55, *p* = 0.16, and *p* = 0.13, respectively, d_f_ = 1 each). All Mospilan treatments were associated with strong effects on mating dance initiation. Following the tarsal treatment with fresh Mospilan residues, fewer than half of the treated males initiated the mating dance (5/11 males; Fisher exact test *p* = 0.007, d_f_ = 1). The effects of tarsal treatment with 24 h-old residues of Mospilan were even more prominent, causing only 5/15 males to initiate the mating dance (Fisher exact test *p* < 0.001, d_f_ = 1). The treatment of the tunnel with the draglines with Mospilan also prevented the males from initiating the mating dance (6/12 males; Fisher exact test *p* = 0.01, d_f_ = 1) (Fig. 2B).

**Figure 3:**
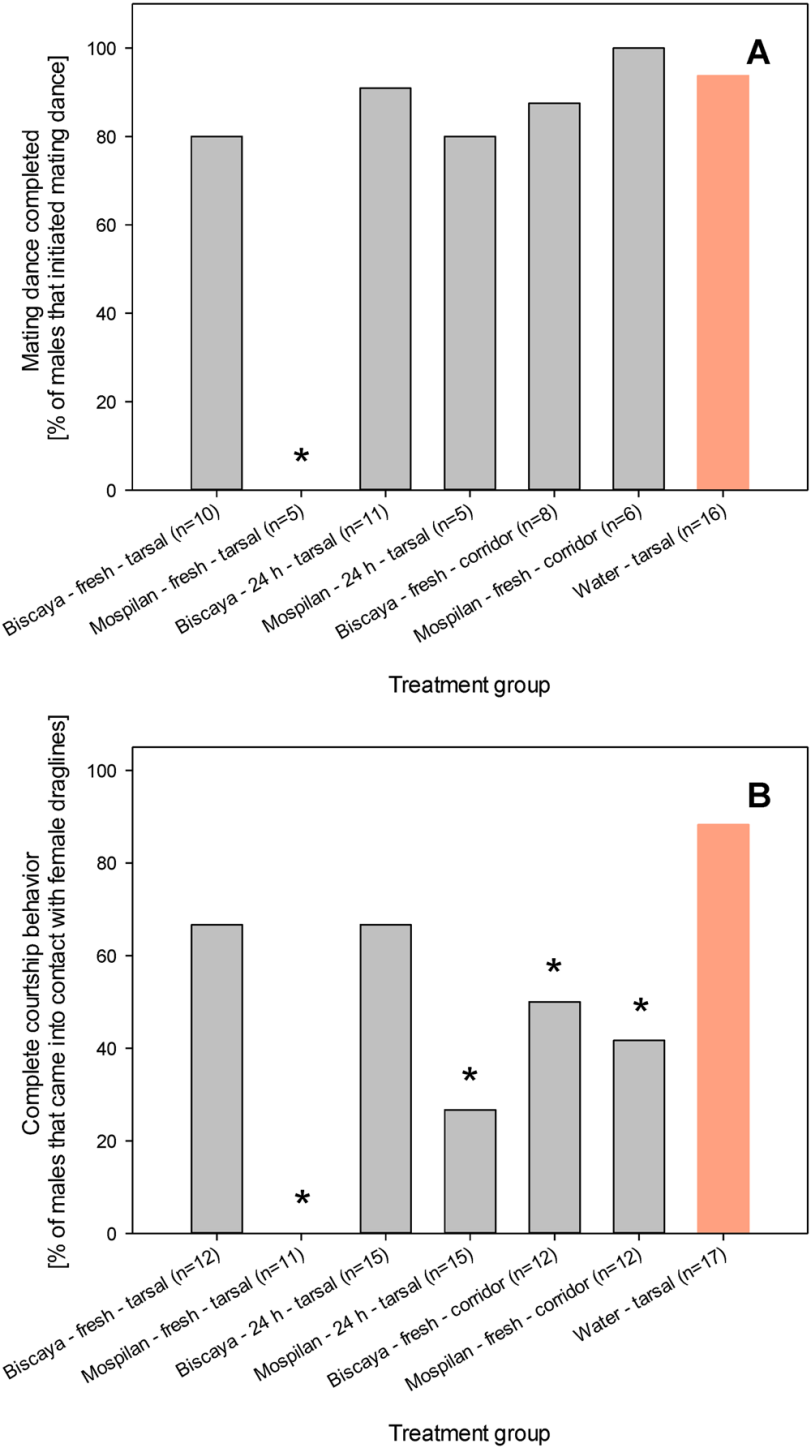
Effects of neonicotinoid insecticides on the completion of courtship behavior in response to contact chemoreception in male *P. agrestis*. Frequencies of males that completed the mating dance in response to contact with female draglines (**A**) and frequencies of males that both chose the tunnel with female draglines and completed the mating dance in response to contact with female draglines (**B**). Numbers of tested individuals in each group are indicated; the provided numbers represent the numbers of males that initiated mating dance (**A**), or the numbers of males that came into contact with female draglines (**B**). Fisher exact test outcomes at *p* < 0.05 are indicated by * when compared to the water-treated group.

Some individuals only initiated the mating dance but did not manage to complete it. However, we did not observe any neonicotinoid-specific effects on the completion of the mating dance except when we applied fresh residues of the neonicotinoids. The tarsal treatment with fresh Mospilan residues prevented the completion of the mating dance in all individuals that initiated this behavior (0/5 males; Fisher exact test *p* < 0.001, d_f_ = 1) (Fig. 3A). Concerning the tarsal treatment with fresh Biscaya residues, the differences were not significant due to the insufficient power of the study.

The males in the control group typically managed to select the tunnel with female draglines as well as complete the mating dance (15/17 males). In the treated groups, many males managed to complete only one of these behaviors but failed to perform another. Therefore, we evaluated the frequencies of individuals that displayed the complete response to the female chemical cues, which was defined as both proper tunnel selection and mating dance completion. In general, the effects of the Biscaya treatments were not significant except for the treatment of the tunnel with Biscaya (8/12 males, 10/15 males, and 6/12 males, respectively; Fisher exact test *p* = 0.20, *p* = 0.21, and *p* = 0.04, respectively, d_f_ = 1 each) (Fig. 3B). All Mospilan treatments were associated with a strong response of the tested males. Following the tarsal treatment with fresh Mospilan residues, male *P. agrestis* were unable to display the full extent of the behavior that is associated with female cue recognition (0/11 males; Fisher exact test *p* < 0.001, d_f_ = 1). The effects of tarsal treatment with 24 h-old residues of Mospilan were similar, but some of the tested males still displayed the full response to female cues (4/11 males; Fisher exact test *p* < 0.001, d_f_ = 1). The treatment of the tunnel with the draglines with Mospilan was the least effective method of Mospilan administration, as it prevented the full response to female cues in only slightly over half of the tested males (5/12 males; Fisher exact test *p* = 0.01, d_f_ = 1) (Fig. 3B).

## Discussion

In the present study, we show that sublethal doses of two major neonicotinoid insecticides that are used for foliar applications in agriculture alter pheromone-guided behavior. These are the first conclusive data regarding the effects of commercially available formulations of neonicotinoid insecticides on the intraspecific chemical communication of spiders. We further provide evidence that the observed effects are not dependent on intense and/or long-term exposure to the neonicotinoids, as would be mimicked by their peroral application or direct contact (dorsal) application. Instead, the one-hour long tarsal exposure to residues of neonicotinoids or even transient exposure to the neonicotinoids that were present in the tunnel surrounding the female draglines were sufficient to induce the observed effects on the courtship behavior of spider males. Immediate manifestation of effects of transient (several seconds-long) exposure to neonicotinoids suggests that the mechanism of action involves either direct modulation of pheromone response or indirect effects mediated by generally toxic effects on olfactory or central neurons. The response to neonicotinoids happens within milliseconds^22–24^; however, only a few cell types can be reached by neonicotinoids within the first few seconds following the exposure^25,26^. The previous studies looked only at effects occurring approximately one hour after oral administration, during direct perfusion of brains or on cell cultures of olfactory neurons. The immediate contact effects observed in the present study were unexpected and further research is needed to elucidate their mechanisms.

Neonicotinoid insecticides are increasingly recognized for their role as information disruptors by modifying the chemical communication system of insects and therefore decreasing the chances of reproduction in target insects^27,28^. The loss of male response to female cues was previously described for a wide spectrum of agrochemicals, such as malathion^29^, deltamethrin^30,31^, beta-cypermethrin^32^, lambda-cyhalothrin^33^, chlorpyrifos^34,35^, as well as the neonicotinoids^22,27,36–39^. In a study that involved the parasitoid wasp *Nasonia vitripennis*, imidacloprid-treated females displayed a suppressed ability to locate the male sex pheromone and were less responsive to the male aphrodisiac than untreated females. Likewise, imidacloprid-treated males were less effective in recognizing females and impaired in terms of perceiving female-derived cuticular hydrocarbons^36^. Neonicotinoids also alter the calling behavior of the tortricid moths *Cydia pomonella*, *Grapholita molesta*, and *Lobesia botrana*^37^. In these species, thiacloprid delayed and reduced the percentage of males responding in a wind tunnel and increased their susceptibility to wind-induced drift^39^. In males of the noctuid moth *Agrotis ipsilon*, clothianidin modulates behavioral sex pheromone responses^27^, which are mediated by antennal lobe output neurons instead of olfactory receptor neurons^22^. In the model of the filth fly *Spalangia endius*, reduced mating competitiveness was observed in males treated with imidacloprid^38^. The effects of neonicotinoids on the courtship behavior of insects can be delayed, and the exposure of larvae can still affect their courtship and mating traits when they develop into adults^40^.

It is interesting that a recent study^41^ reported feeding deterrent behavior in the same spider species in response to prey that is contaminated by neonicotinoids. The authors, however, did not observe the spiders to be deterred from prey killing but from prey consumption only. They suggested that the first contact with neonicotinoids during prey capture had already affected the health of the spiders. In the present study, we used the maze, where the spiders became first in contact with neonicotinoids and only then had to decide, which of the two neonicotinoid-treated corridors to use, whether the one that contained the silk (extending to the choice area) or another without the silk (Fig. 1). It is known that contact with neonicotinoids often leads to the temporary paralysis of spiders^18^. Therefore, a follow-up study needs to incorporate a contactless version of the maze used, in which the spiders will have no contact with the neonicotinoid residues despite their application to inaccessible parts of the maze. We speculate that physical contact is necessary to observe both deterrent effects on behavior and the changes in male courtship behavior described here. Despite the fact that contact is likely necessary, we have shown that even transient, short-term exposure to the neonicotinoids that were present only in the tunnel with female draglines was sufficient to induce the full extent of observed effects that hampered male courtship and mating traits.
